# The online measured black carbon aerosol and source orientations in the Nam Co region, Tibet

**DOI:** 10.1007/s11356-017-0165-1

**Published:** 2017-09-17

**Authors:** Xin Zhang, Jing Ming, Zhongqin Li, Feiteng Wang, Guoshuai Zhang

**Affiliations:** 1 Northwest Normal University, Lanzhou, 730070 China; 2 Biogeo/Multiphase Chemistry Department, Max Planck Institute for Chemistry, 55128 Mainz, Germany; 3 State Key Laboratory of Cryospheric Sciences, Chinese Academy of Sciences, Lanzhou, 730000 China; 4 Institute of Tibetan Plateau Research, Chinese Academy of Sciences, Beijing, 100101 China

**Keywords:** Equivalent black carbon (eBC), Air pollution, Aerosol optical depth (AOD), Nam Co, Tibet

## Abstract

Equivalent black carbon (eBC) mass concentrations were measured by an aethalometer (AE-31) in the Nam Co, central Tibet from 2010 to 2014. Different from previous filter-sampling studies (Ming et al., J Environ Sci 22(11):1748–1756, 2010; Zhao et al., Environ Sci Pollut Res 20:5827–5838, 2013), the first high-resolution online eBC measurement conducted in central Tibet is reported here, allowing to discuss the diurnal variations as well as seasonal variabilities of eBC. Average daily eBC concentration was 74 ± 50 ng/m^3^, reflecting a global background level. Meteorological conditions influenced eBC concentrations largely at seasonal scale, which are higher in February–May but lower in June–January. The highest eBC concentrations (greater than 210 ng/m^3^) were more associated with the W and WSW winds smaller than 6 m/s. The diurnal variations of eBC showed plateaus from 10:00 to 15:00 with seasonal variations, associated with local anthropogenic activities, such as indigenous Tibetan burning animal waste and tourism traffic. The PBLHs showed a co-variance with eBC concentrations, implicating close sources. The aerosol optical depths derived from the MODIS data over the Nam Co Observatory Station (NCOS)-included sub-area (30° N–40° N, 90° E–100° E) showed significant relationship with eBC concentrations. This suggests that nearby or short-distance sources other than long-distance transported pollutants could be important contributors to eBC concentrations at the NCOS, different from the conclusions suggested by previous studies.

## Introduction

Carbonaceous aerosols, which primarily consist of black carbon (BC) and organic carbon (OC), impact on global climate and environment largely. eBC is produced mainly from incomplete combustion of biomass and fossil fuels, which is the second most important agent of global warming after CO_2_ (Bond et al. 2013; Panicker et al. 2010). In the atmosphere, BC particles strongly absorb visible light and emit infrared radiation and therefore causes ambient air being heated (Chung and Seinfeld 2002; Panicker et al. 2013). BC deposited on ice and snow surfaces can reduce surface albedo by absorbing more solar radiation, which may accelerate glacier melting and result in change in glaciers (Ming et al. 2009; Santos et al. 2014; Ming et al. 2015; Ming et al. 2016). eBC not only plays a major role in climate but also causes negative influences on human health (Bond et al. 2013; Ramanathan et al. 2007; Mordukhovich et al. 2009).

The Tibetan Plateau (TP) adjoins several major BC emission regions, such as South Asia (e.g., India) and East Asia (e.g., China). Many previous researches focused around urban and rural BC measurements in China, and India institutions did numbers of studies on regional air pollution (e.g., Babu et al. 2002; Tripathi et al. 2005; Praveen et al. 2012; Song et al. 2013; Cao et al. 2009; Gao et al. 2014; Huang et al. 2011; Surendran et al. 2013). However, BC studies are still very scarce to date in high-altitude or remote regions, such as Tibetan Plateau.

The TP with a vast area of seasonal snow cover and glaciers is remote from populated regions and provides ideal locations for monitoring BC at global background level (e.g., Zhang et al. 2007; Cong et al. 2009a; Ming et al. 2010). To improve the understandings of atmospheric backgrounds, China has been endeavored to measure BC since the built of the Mt. Waliguan Observatory (36.17° N, 100.54° E, 3810 m a.s.l.) in 1994, a baseline station of the Global Atmospheric Watch (GAW). Ever since then, more and more BC measurements have been carried out at regional background observatories. For example, Zhao et al. (2012) used an aethalometer (AE-31) to measure the eBC concentrations and suggested the eBC background concentration in Qilian Shan between 18 and 72 ng/m^3^ with the highest in summer and the lowest in autumn. Ming et al. (2010) collected filter samples in Nam Co during July of 2006 through January of 2007 and estimated that the average BC concentrations were 82 ng/m^3^ through the thermos/optical reflectance (TOR) method. Using the same methods, the BC concentration was found at 13–45 ng/m^3^ from July 2006 to December 2009 at Nam Co Observatory Station (NCOS) (Zhao et al. 2013). In these studies, BC was collected onto filters by air samplers and taken back to laboratory analysis. Offline measurements could better understand physical characteristics, chemical characteristics, and biochemical characteristics of BC. One disadvantage of this approach is that it requires a lot of human resource at a remote area and gets a lower temporal resolution. Online measurements are critical to characterize short-term variabilities in BC, such as measuring the diurnal variations of BC and sourcing emissions that vary rapidly.

Previous studies basically focused on the temporal variations and chemical characteristics through offline measurements with much coarser time resolutions in Nam Co (e.g., Ming et al. 2010; Zhao et al. 2013). However, around one filter sample per week in previous studies would not allow to analyze the diurnal variations of BC. What is more, recent variations of BC remain unknown, although the online BC observation at the NCOS has been built up since 2010. In this work, a high temporal resolution data of equivalent BC (eBC) concentration measured by an aethalometer instrument (Model AE-31) at the NCOS are firstly presented over Tibet to our knowledge. The concept of eBC comes from a previous study (Petzold et al. 2013). Petzold et al. (2013) made the most recent attempt to determine more precise definitions for quantitative study of BC, making terminology recommendations based on specific measurement techniques. Monthly and seasonal variations of eBC concentrations will be discussed when we present the temporal characteristics of eBC in the most recent time period. Diurnally eBC-varying patterns in different seasons will be firstly reported here. Furthermore, the relationships between eBC, meteorological conditions, and aerosol optical depth (AOD) will be used to investigate the impacting factors on local eBC variations. Finally, the potential sources and transport pathways of eBC to the site will be discussed.

## Instrument and methodology

### Site description and local meteorology

**Fig. 1:**
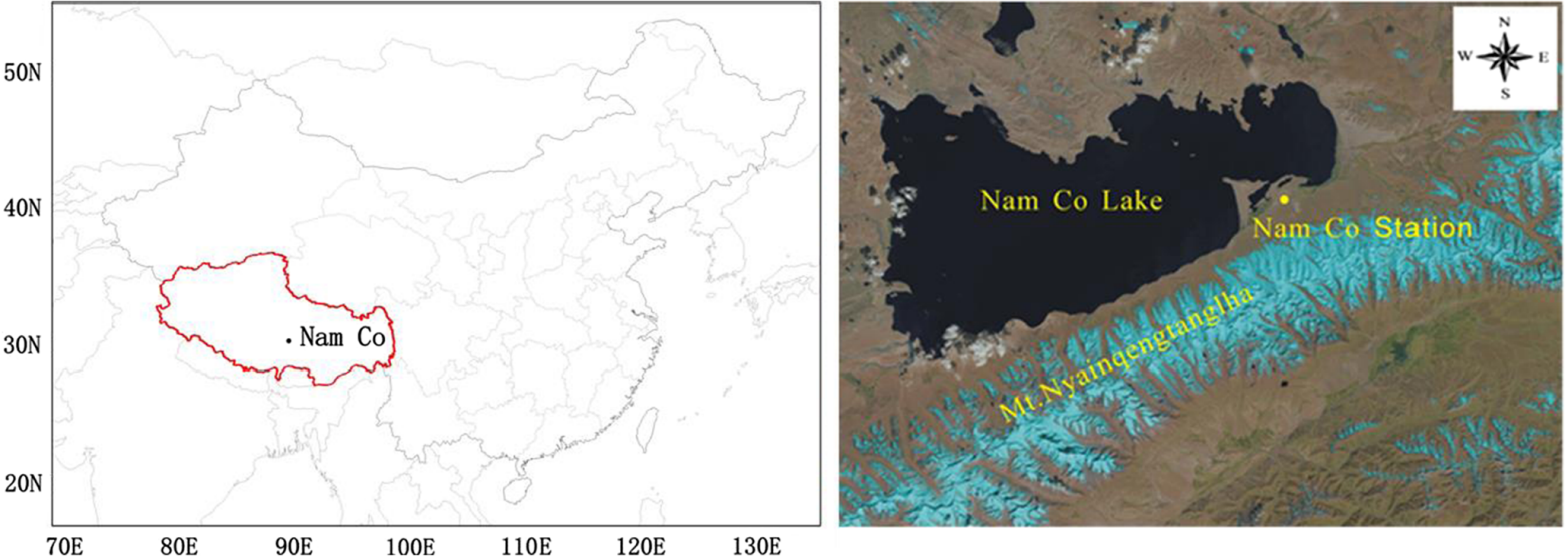
Location map of the NCOS (30° 46′ N, 90° 59′ E, 4730 m a.s.l.)

The NCOS locates on the southeast shore of the Nam Co Lake and the north foot of Mt. Nyainqentanglha (Fig. 1). The meteorology (temperature, relative humidity, wind, precipitation and etc.) has been continuously monitored by an automatic weather station (AWS). More detailed introductions of the meteorology measurement setup can be read in previous studies (Ming et al. 2010; Zhao et al. 2013). There are lightly anthropogenic emissions due to grazing and touring activities a few or tens of kilometers away. Earlier studies conducted here showed that BC concentration level could reflect weak anthropogenic disturbances (Ming et al. 2010; Wan et al. 2015).

**Fig. 2:**
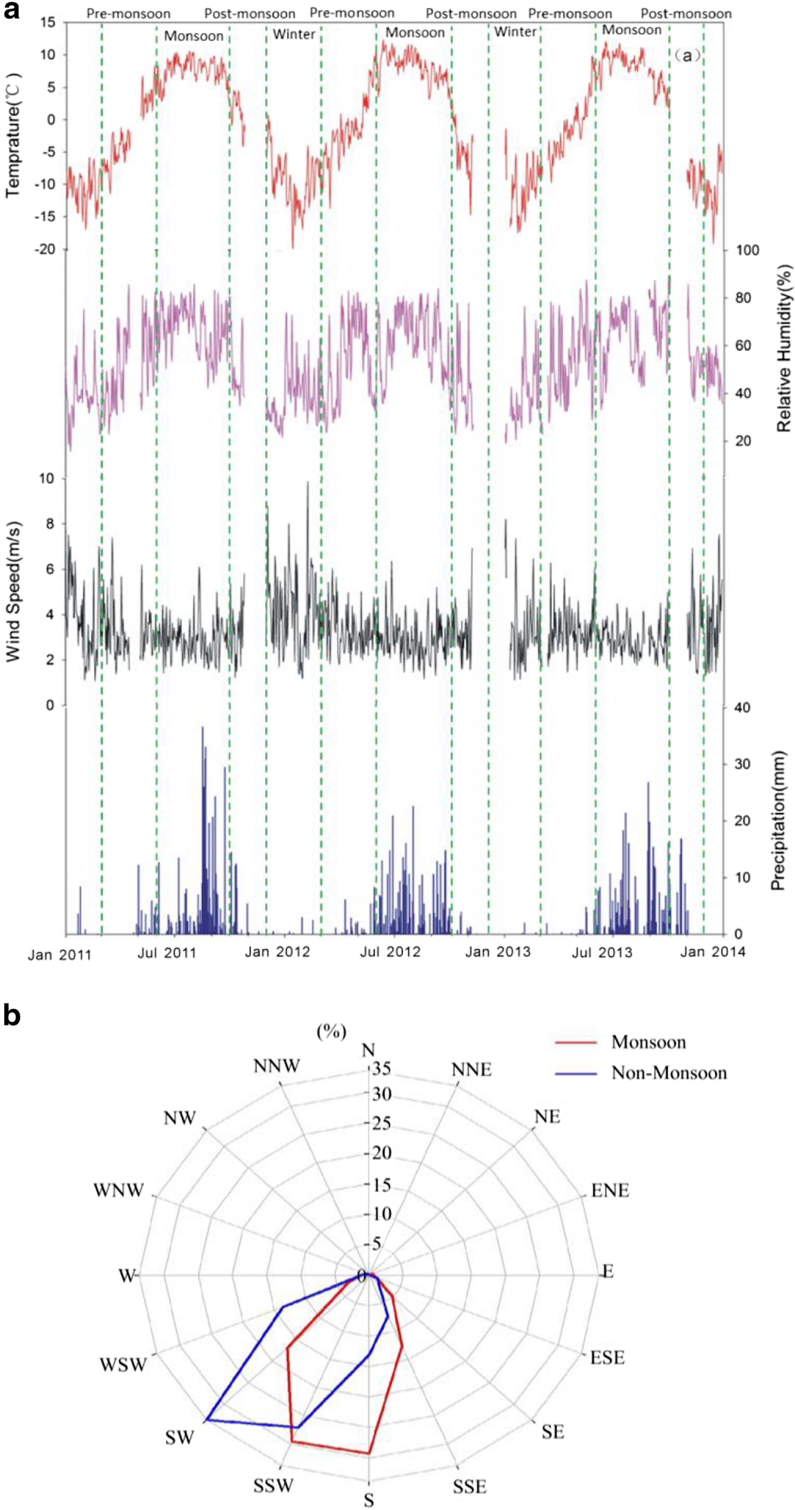
**a** Elementary meteorological observations at the NCOS from December 2010 to January 2014, including air temperature, RH, wind speeds, and precipitations. **b** The distributions of monsoon and non-monsoon wind directions and their individual percentages

Daily averaged air temperature, relative humidity (RH), wind, and precipitation are shown in Fig. 2. Meteorological conditions at the NCOS showed obvious seasonal variations. The daily mean air temperature during June–September varied from 6.2 to 9.1 °C; while during October–May, it ranged from − 11.8 to 3.3 °C. Precipitations were mainly observed in monsoon seasons (June to September). The westerlies dominated in non-monsoon seasons and led to fewer precipitations. The site is dry during non-monsoon seasons (November to May) with the mean RH below 45%. The highest RH above 60% occurred during monsoon seasons (June to September) with the prevailing southern winds from the Indian Ocean. Throughout the sampling period, wind speeds varied from 1.1 to 9.9 m/s; the daily average wind speed was 3.3 m/s. During monsoons, the NCOS site is majorly influenced by the Indian monsoon, which brings humid air mass of Indian Ocean from SSW (29.5%), S (29.3%), and SW (16%). During non-monsoons, the NCOS site is mainly dominated by drier westerly winds, which results in less precipitations. The prevailing wind directions were SW (33.6%), SSW (27.1%), and WSW (13.6%).

### Instrument and measurement

Real-time and continuous measurements of eBC were carried out from October 2010 to October 2014 using a seven-channel aethalometer (Model AE-31, Magee Scientific®, USA). Optical attenuation-detecting technique is used in the aethalometer. The mass concentration of eBC is calculated from an incremental attenuation between two measurements using the effective specific mass absorption cross section (MAC) of the eBC deposited on the filter, area of the sample spot, and the flow rate. The inlet without cutoff amounted of the aethalometer sucked the ambient air directly from 4 m above the ground level at a flow rate of 4 LPM, and measurements were made every 5 min. Periodical calibrations and flow check of the instrument were carried out to maintain the data reliability and precision. This instrument was calibrated in 2011 and 2013 by the manufacturer, Magee Technologies™. A previous study argued that the AE-31 instrument needs to be corrected for the artifacts inherent in the filter-based technique (Collaud Coen et al. 2010), which was not applied for our instrument and should be addressed here. BC is the dominant absorber at 880 nm with the imaginary part of refractive index greater than 0.44, which is two orders higher than that of OC and dust (Lubin et al. 2002; Begam et al. 2016; Wang et al. 2016). We used the 880-nm channel to estimate eBC using the laboratory calibrated value (16.6 m^2^/g) as MAC. The results could be influenced by OC, although 880 nm was reported not to be the radiation-absorbing wavelength of OC (Kirchstetter et al. 2004; Ramanathan et al. 2011). Cong et al. (2009b) conducted a one-year aerosol particle sampling and analyzed the individual particles in the samples, indicating that majority (greater than 80%) of the particles in number are soot (aggregates or individuals, ~ 20%) and aluminosilicates/quartz (non-absorptive dust, ~ 60%) in all seasons. Other absorptive Fe-oxide dust comprised less than 10% of total number concentration, so we presume here that all absorptive contributions were from black carbon.

The manufacturer of the aethalometer gives an error less than 5% in the eBC concentration measurement (Hansen 2005). However, the aethalometer may produce negative values in low-concentration sampling conditions and at a high time resolution. Filter-based optical attenuation technique including shadowing and multiple-scattering effects can contribute up to 30% of uncertainty in lower concentrations and at a high time resolution (Hagler et al. 2011). The optimized noise reduction averaging (ONA) algorithm has been used to post-process the negative values from real-time eBC of aethalometer (see the details in Hagler et al. 2011). The ONA algorithm is used to calculate variable averaging time intervals based on a default minimum attenuation increment (ΔATN_min_ = 0.05) to reduce the noise in the eBC attenuation data. The ONA algorithm leads to significant noise reductions and much more reasonable temporal variations in aethalometer data (Cheng and Lin 2013; Park et al. 2010).

### Uncertainty of eBC arising from the absorption by mineral dust

To exactly quantify the separate absorbing proportions by black carbon and mineral dust is still in debate. Some studies suggested the absorption from dust when measuring eBC by an aethalomter cannot be neglected (e.g., Fialho et al. 2005; Yang et al. 2009; Schauer et al. 2016, while there are still strong arguments against this view that the absorption by dust could not be comparable to that by BC (e.g., Hansen et al. 1993; Schnell et al. 1994; Bodhaine 1995).

The scientific base of neglecting the absorption of dust when measuring with an aethalometer is that the primary absorbing component in dust, hematite (Fe_2_O_3_), has ~ 1/200 of absorption equivalent to that of BC (Bodhaine 1995). During a large dust storm recorded at Mauna Loa in 1991, it was found a maximum of ~ 20% absorption contributed by dust (Schnell et al. 1994). Hansen et al. (1993) found that optical absorption due to dust averaged approximately 11% in a central Asian desert in 1989. And these results were not mentioned in the “favoring dust absorption” studies.

At the NCOS site, in particular, the primary aerosol particles in number concentrations are quartz and aluminosilicates (non-absorptive, ~ 60%) and soot (absorptive, ~ 20%), but not hematite (less than 10%) (Cong et al. 2009b). Taking both the small quantity of absorptive dust and its weakly absorptive nature compared to black carbon into account, we presumed that the majority of absorption was due to BC, neglecting the possible absorption by dust in this study. The estimated uncertainty from dust involved in eBC could be ~ 0.25% (~ 1/200/2 × 100%). This presumption could be finitely reasonable in this remote site of the Tibetan Plateau.

### Backward trajectory analysis

Five-day air mass backward trajectories were calculated by the hybrid single-particle Lagrangian integrated trajectory (HYSPLIT) model (Draxler and Hess 1998) to determine the potential transport pathways of eBC. The model elevation at the NCOS is ~ 500 m lower than the true elevation, and we took the NCOS site as the end point setting the height at 500 m above ground level (AGL) starting at 00:00 (Beijing time) on each day from October 1, 2010 to October 31, 2014. The meteorolgocial data used for trajectory calculating were the Global Data Assimilation System (GDAS) data (1° × 1°) and downloaded from the web sever of NOAA Air Resources Laboratory (Zha et al. 2014; Calvello et al. 2010; Jung et al. 2010).

## Results and discussions

### Variations of eBC concentrations

**Fig. 3:**
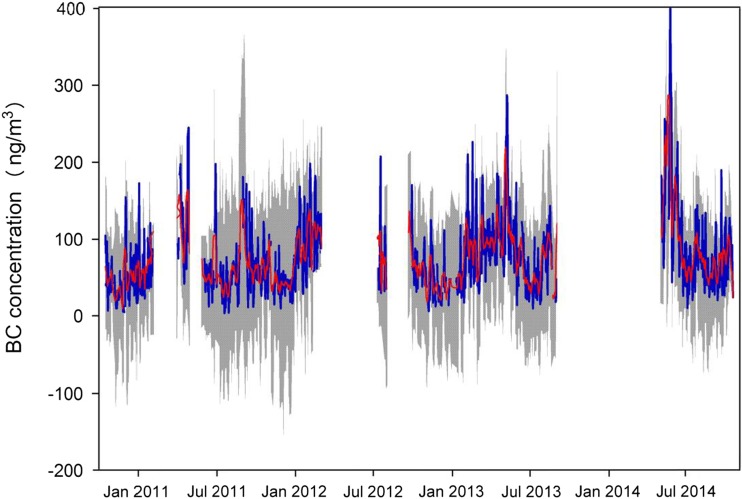
Variation of daily eBC concentrations during the study period. The *blue lines* indicate daily eBC concentrations; *gray areas* indicate the ± 1σ standard deviations; the *red lines* indicate the 7-day moving averages, and the *black lines* indicate the period trends

The daily mean variations of eBC concentrations at the NCOS from October 2010 to October 2014 are shown in Fig. 3. Daily mean eBC concentrations are substantially in the range of 3–417 ng/m^3^ with a mean of 74 ± 50 ng/m^3^. The highest daily eBC concentration occurred on May 23, 2014 (417 ng/m^3^), while the lowest was on July 19, 2011 (3 ng/m^3^). The high values of eBC observed could be attributed to active biomass burning and relatively drier weather (Zhao et al. 2013). The lowest eBC values may be related to frequent precipitations acting as a significant scavenging factor during monsoon seasons (Ming et al. 2010). Total 107 peak values were higher than 125 ng/m^3^ (mean daily eBC concentration + 1σ). Peaks (58%) occurred on May and June in 2014, and 27% peaks occurred on April 2011, February 2012, and May 2013, which belong to non-monsoon seasons (November–May).

The eBC concentrations at the NCOS could be representing a global background, defined as concentration in well-mixed atmosphere at global scale. The 7-day moving average varied between 14 and 286 ng/m^3^. During the study period, the eBC concentration was close to that of Mt. Waliguan (130–300 ng/m^3^) and Qilian Shan (50–120 ng/m^3^) (Tang et al. 1999; Zhao et al. 2012).

**Fig. 4:**
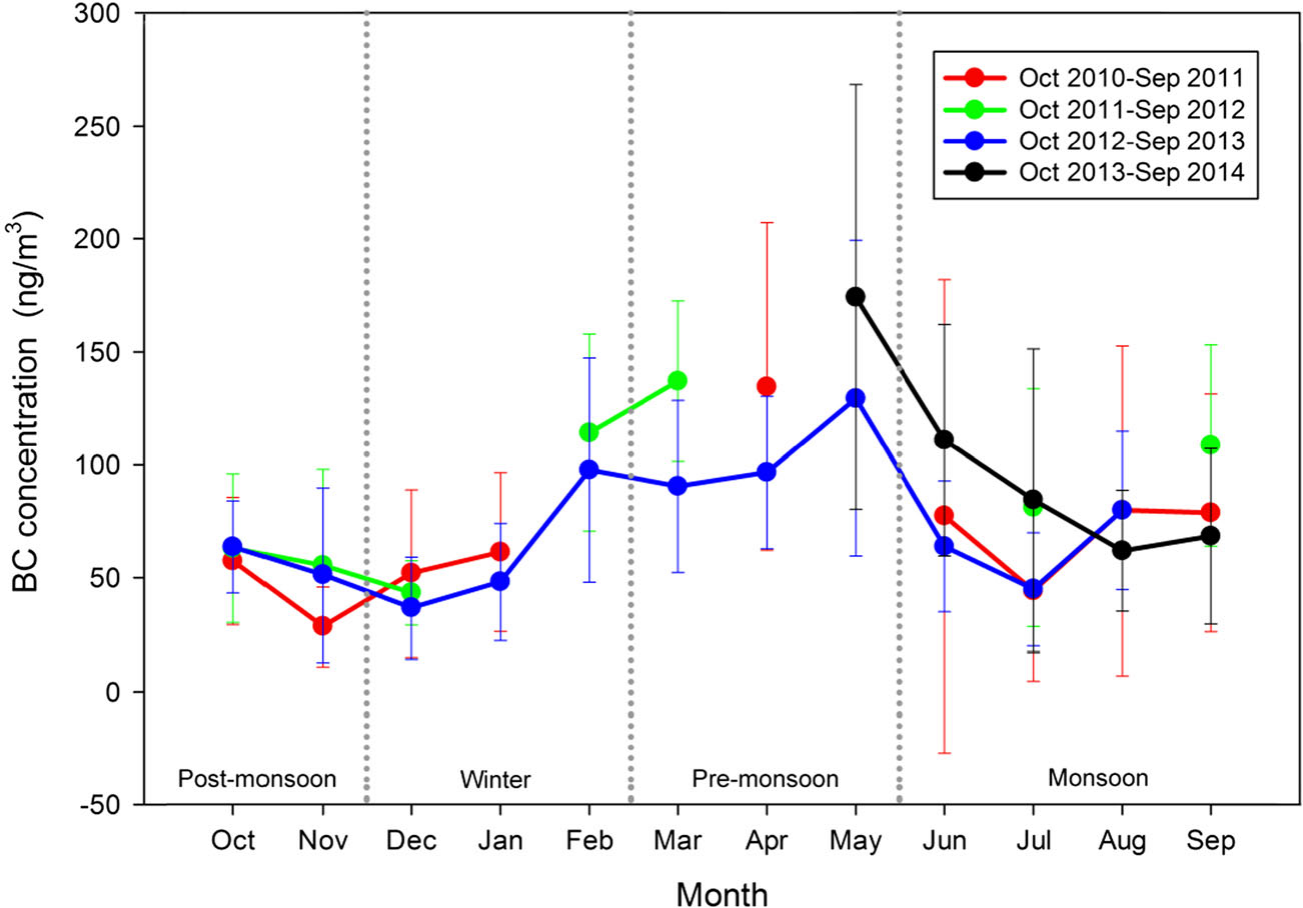
Monthly variations of eBC (with ± σ) at NCOS during the study period

Monthly mean variations of eBC concentrations during the study period are shown in Fig. 4. eBC monthly mean concentration at the NCOS varied between 43 and 173 ng/m^3^. The highest monthly mean occurred in May 2014, while the lowest monthly mean eBC was in November 2010. The highest concentration might be related to several episodic pollutions. For example, the daily averaged eBC concentration was higher than 200 ng/m^3^ from May 20 to May 25.

The seasonally averaged eBC values during the study periods are 127, 77, 56, and 75 ng/m^3^ for pre-monsoon, monsoon, post-monsoon, and winter, respectively (Fig. 4). The pre-monsoon seasons (March to May) have the highest mean eBC concentration, followed by monsoons, post-monsoons, and winters. The high eBC concentrations in pre-monsoon could be attributed to meteorological conditions such as smaller wind speeds and fewer precipitations (Fig. 1). The effects of meteorological conditions will be further explained in section 3.4. The low values of eBC in monsoons are associated with more precipitations although biomass burnings and tourist activities are also more intensive.

eBC concentrations have obvious seasonal cycles (Fig. 4). However, the eBC varying pattern is different from those reported in Lhasa and other urban/rural sites of South Asia, where consistently higher eBC values were observed during winters and lower values during monsoons (Praveen et al. 2012; Ningombam et al. 2014). A reasonable explanation for the difference could be that direct biofuel burning (heating, cooking, etc.) of city residents in the winter induces higher eBC concentrations; correspondingly, the seasonal variations of eBC here are similar to those observed at the NCO-P site (Marinoni et al. 2010) and Mt. Waliguan (Tang et al. 1999), showing higher eBC concentrations in pre-monsoons and lower in monsoons.

### Possible long-distance transport pathways of eBC

**Fig. 5:**
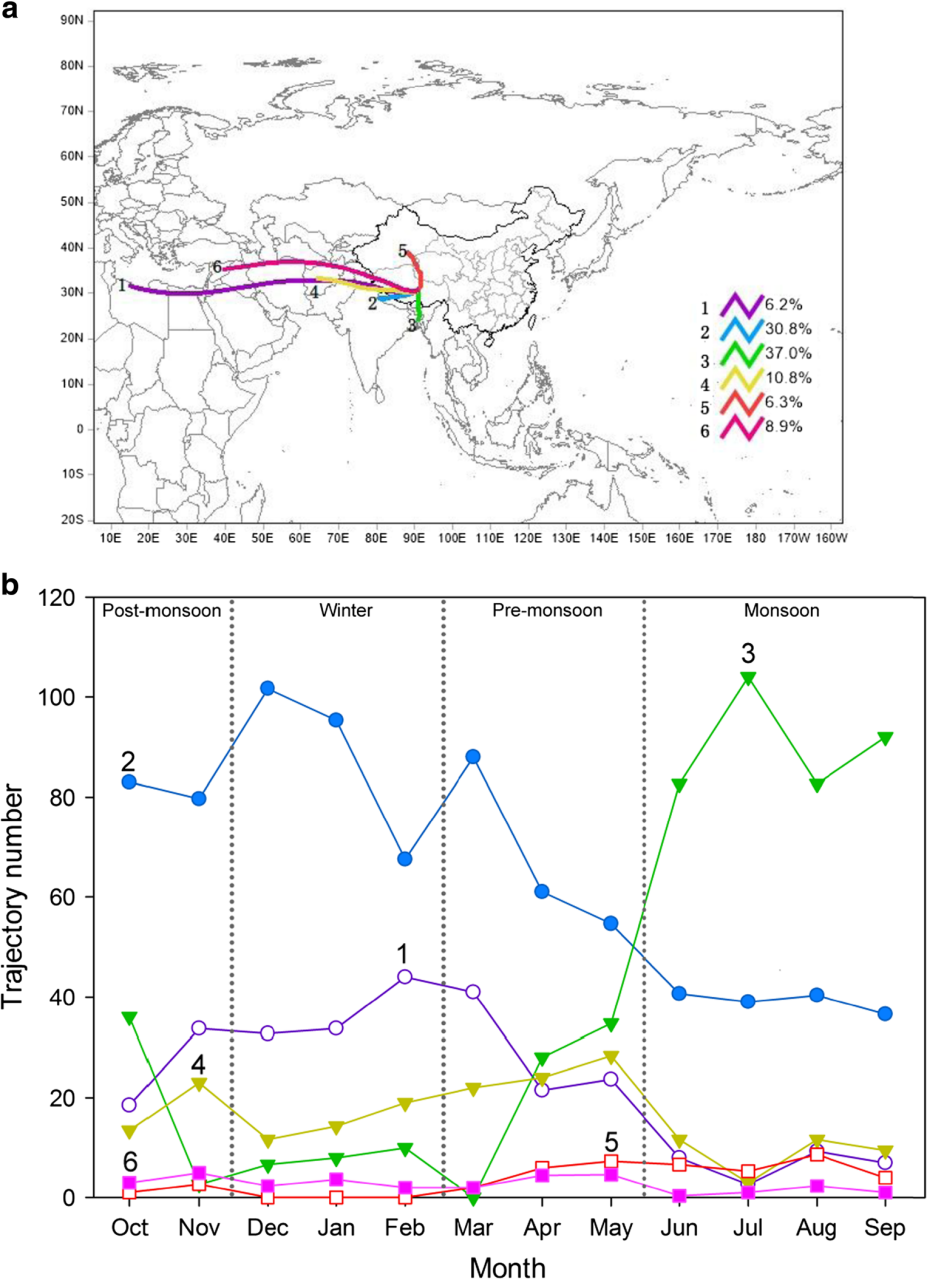
**a** Cluster mean 5-day backward air mass trajectories calculated by the HYSPLIT model ending at the NCOS including the percentages of each cluster trajectories. **b** Monthly variations of the relative trajectory numbers

To determine the potential long-distance transport pathways of air masses reaching the NCOS, five-day backward air mass trajectories are calculated using the HYSPLIT model and the NCEP/NCAR GDAS dataset (Draxler and Rolph 2003). The model ran every 6 h throughout the sampling period ending at the point of 500 m above the modeled NCOS topography. We obtained a total number of 967 daily trajectories averaged from ~ 4500 6-h trajectories, which can be grouped into six clusters via the built-in clustering tool in the model. We also calculated median trajectory for each cluster. More details of the cluster analysis are available in the literatures (Dumka et al. 2010; Joshi et al. 2015). Figure 5a shows that there are six pathways for air masses to arrive at the NCOS from their distant sources. Trajectories from Bangladesh (37%) and Northern India (31%) account for 68% of the total daily trajectories, indicated by mean trajectories 2 and 3, respectively (Fig. 5a). In monsoons, air masses were mostly from the Bangladesh (trajectory 3), indicating that emissions in South Asia could travel to the central TP by the India monsoon (Fig. 5b). The findings are corresponding to previous studies (Zhao et al. 2013; Cong et al. 2015; Zhang et al. 2015). However, non-monsoon air masses were mainly from Northern India (trajectory 2) and West Asia, and trajectories from the west and north account ~ 30% of the total trajectories. We presumed that dust from the north and west directions does not contribute significantly to the total absorption as black carbon does, based on the considerations mentioned in section 2.2. The TP is a relatively clean region with weak emissions, while it is also surrounded by the world’s two strongest BC emitting regions, South Asia and East Asia who account for ~ 40% of global emissions (Bond et al. 2013).

Table 1 lists the statistics of trajectory numbers and eBC concentrations during the sampling period. The pollution events are defined as peak values of eBC concentrations (greater than 125 ng/m^3^, i.e., mean + 1*σ*). The peak values mainly occurred in trajectory 2 (27.1%) and trajectory 3 (35.5%), but had lower mean eBC concentrations than trajectory 4, trajectory 5, and trajectory 6. The probable reason is that the trajectory 3 influenced by India monsoon during monsoon and precipitation played a key role in scavenging of BC (Zhao et al. 2013). Trajectory 4 and trajectory 6 were influenced by drier westerlies. The occurrences of *P*_eBC_ and major trajectory numbers suggest the influence of South Asia emissions on the NCOS by monsoons.

**Table 1:** Statistics of trajectories and eBC concentrations during the study period

Trajectory code	Trajectory number	MEAN_eBC_ (ng/m^3^)	PT number	*P*_eBC_ (ng/m^3^)
1	60	70 ± 40	4	165 ± 32
2	298	70 ± 48	29	170 ± 44
3	358	70 ± 49	38	168 ± 54
4	104	88 ± 60	16	188 ± 56.34
5	61	86 ± 48	9	161 ± 46.70
6	86	82 ± 57	11	184 ± 69
All	967	74 ± 50	107	172 ± 52

MEAN_eBC_ is the mean value of eBC concentrations. Peak trajectory (PT) number is the number of trajectories when *P*_eBC_ is detected. *P*_eBC_ is mean BC peak (greater than 125 ng/m^3^, i.e., mean + 1*σ*)

### Impacts of meteorological conditions on eBC variations

**Fig. 6:**
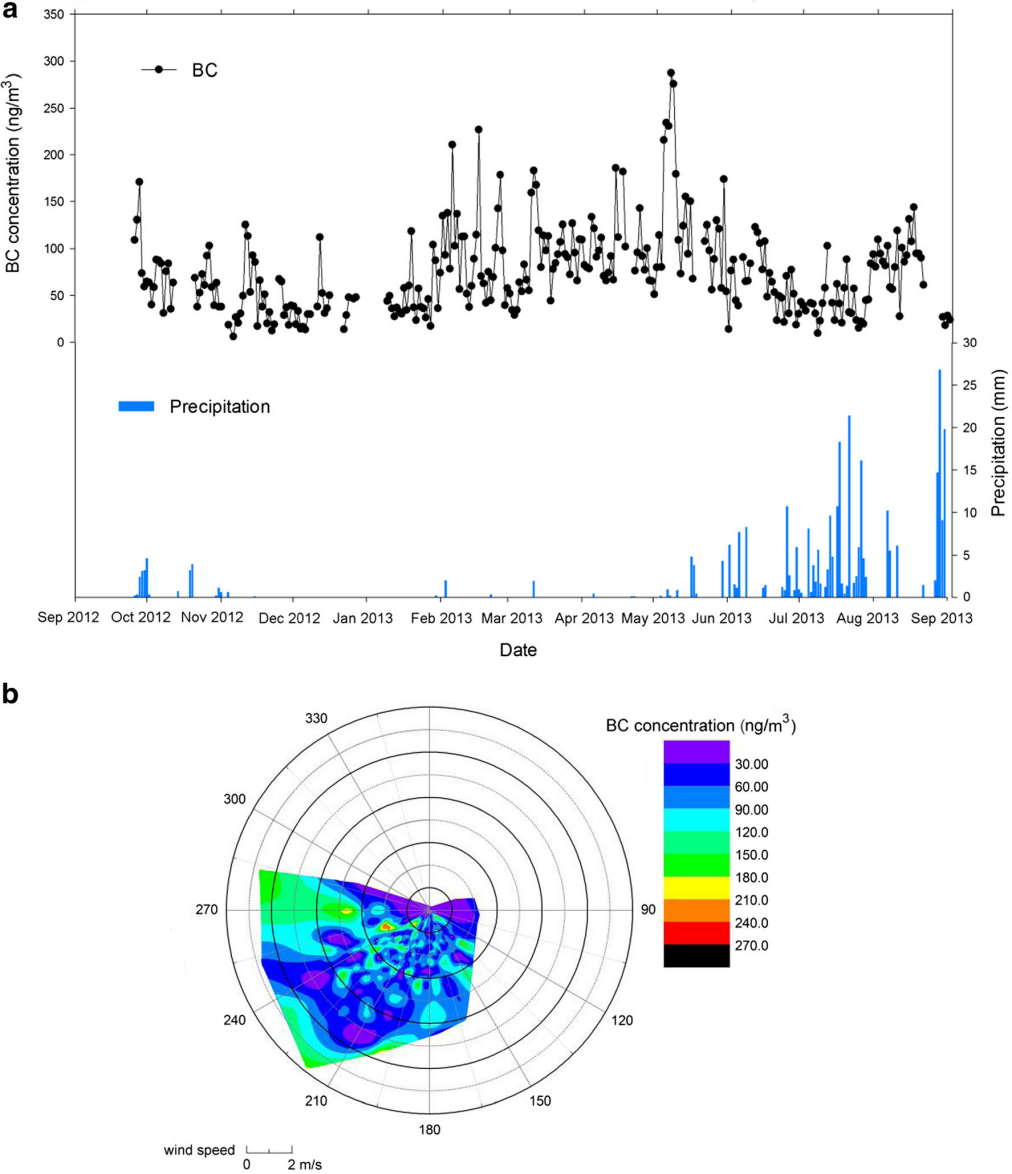
**a** eBC concentrations and precipitations during September 2012 to September 2013. **b** Radar map of average eBC and winds during the study period

Precipitations and winds are also significant factors of BC concentrations in the Nam Co region (Ming et al. 2010; Zhao et al. 2012). The lower eBC concentrations in Monsoons (June to October) were associated with precipitations. Compared with monsoon seasons, pre-monsoon seasons have higher eBC concentrations and less precipitation (Fig. 6a). Figure 6b shows the relationship between eBC and winds at the NCOS. The eBC is higher, while W and WSW winds prevail and lower when SSW and SW prevail. Higher eBC concentrations mostly occurred when the wind directions were generally western, and some are higher than 210 ng/m^3^. This indicates that the transports by prevailing westerlies could bring extremely high eBC concentrations. However, when wind speeds were larger than 6 m/s from other directions, eBC concentrations were generally smaller than 60 ng/m^3^, suggesting the dispersion effects of high-speed winds. The variations of wind speeds and eBC concentrations indicate that eBC here could be largely due to short-distance emissions, but not to well-mixed air masses from long-distance transports.

**Fig. 7:**
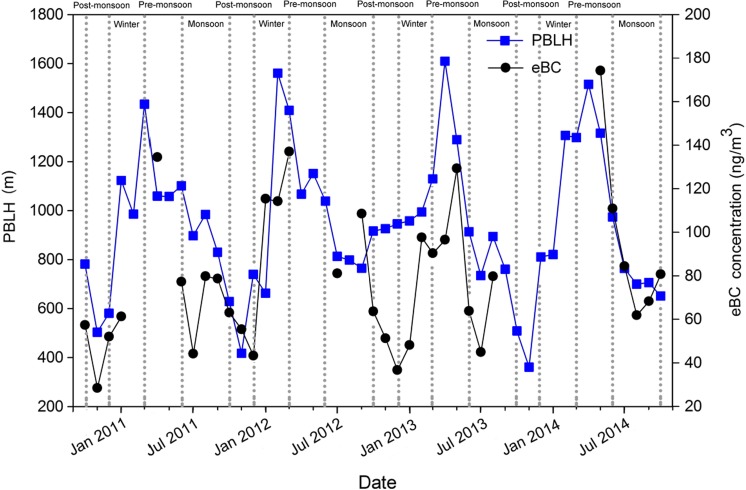
The co-variation of monthly PBLH and eBC concentrations during the study period at the NCOS

The monthly variations in eBC were associated with the planetary boundary layer height (PBLH) over the NCOS. Figure 7 shows the PBLH variation at NCOS in the 1° × 1° grid (30° N–31° N, 90° E–91° E) during the sampling period downloaded from GDAS dataset of the NOAA (http://www.arl.noaa.gov/ready.php). The PBLH had a strong monthly variation and showed the highest in April 2013 and the lowest in November 2013. The evolution of PBLHs depends on solar radiation heating, and thus, the shallow PBLH in November is likely due to the limited radiation time. The positive correlation is (*R* ^2^ = 0.33) between eBC concentrations and PBLHs during 2010 to 2014, although was not very close. In most time, eBC and PBLH show co-variations, except the period of late 2012 to early 2013, and PBLH and eBC had a much stronger correlation (*R* ^2^ = 0.88, *α* = 0.01) in 2014. Normally, PBLH and eBC concentrations have significant negative correlations in urban areas due to the compressed effect when PBLHs are thinner with lower temperatures and stable sources during nighttime and BC mass concentrations would be high with the deepening process of local PBLH, e.g., Beijing (Guinot et al. 2007). Whereas, the eBC-PBLH co-developing pattern here implicates inflowing eBC from closer sources associated with local boundary layer convections.

### Diurnal variations of eBC concentrations

**Fig. 8:**
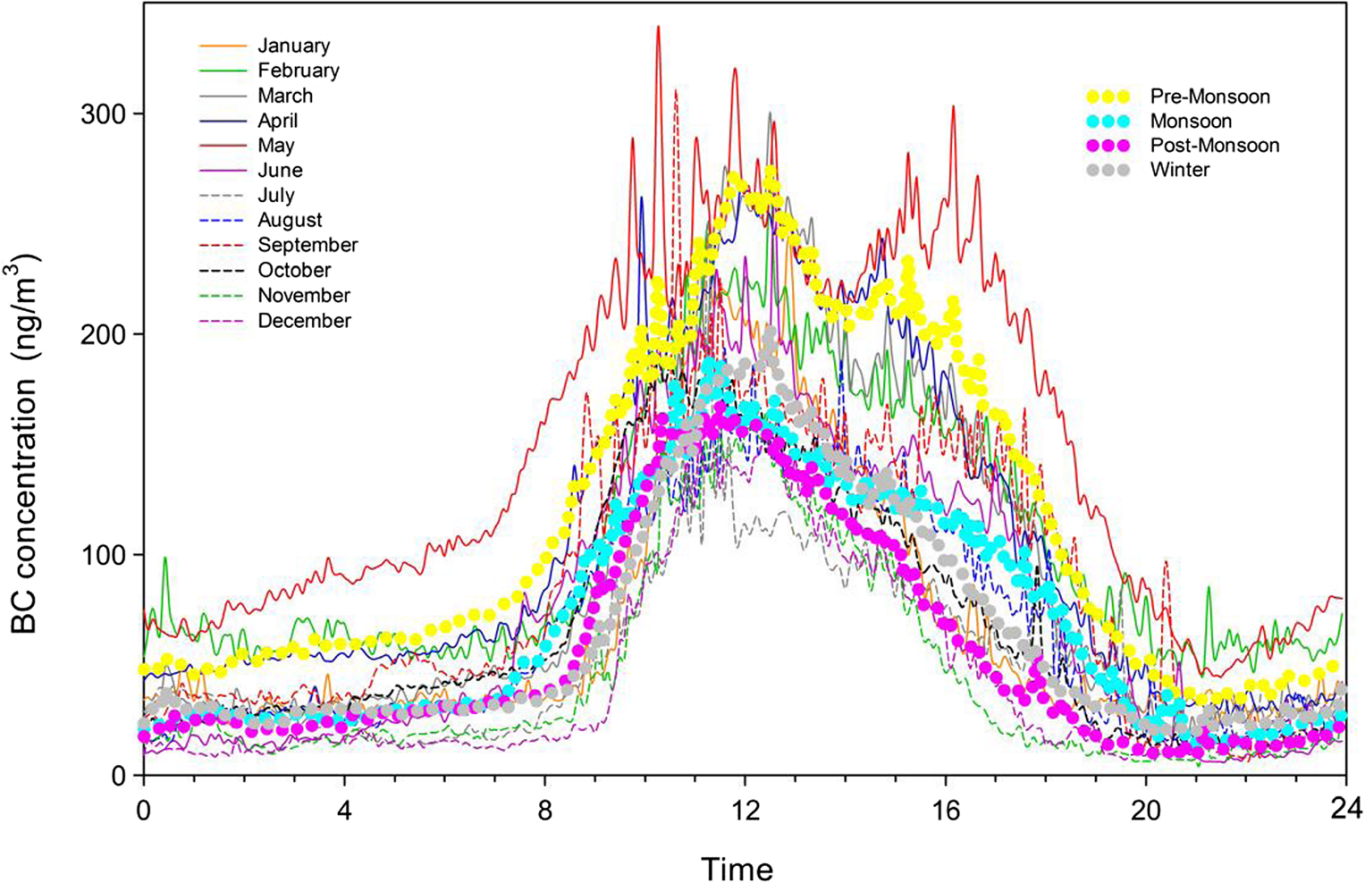
Diurnal variations of eBC concentrations during the study period at NCOS

The analysis on the diurnal variations of eBC is a useful approach to study the effects of mesoscale atmospheric processes and local human activities on eBC (Dumka et al. 2010; Gadhavi and Jayaraman 2010). Figure 8 shows the monthly and seasonally mean diurnal variations of eBC. eBC concentrations had similar peaks shown at ~ 10:00 to ~ 15:00 (Beijing time), which is according to the active time of local residents (cooking and heating using yak’s dry dung) and tourists (traffic and other biofuel burnings). The mean eBC in the daytime (8:00 to 20:00) was higher than at nighttime (20:00 to next 8:00), strongly indicating the result of short-distance or nearby anthropogenic activity sources. The eBC concentrations start rising gradually from 08:00 to 11:00. The maximum concentrations usually occur at noontime and then decrease until 16:00 and keep low and horizontal to next morning. The morning and nighttime eBC concentrations during the pre-monsoons are much higher than other seasons, and they start increasing ~ 1 h earlier than other seasons probably due to earlier sunrise leading to earlier more active anthropogenic activities and involving more influence from the boundary layer and more polluted regions. The diurnal variation of eBC at the NCOS was similar to the research at Ranwu, Tibet, which showed the diurnal pattern featured with a peak shortly after sunrise and a decrease before noon; then, the eBC concentrations remain flat during the nighttime and then reach a minimum before sunrise (Wang et al. 2016).

### Aerosol optical depth and its relationship with eBC

**Fig. 9:**
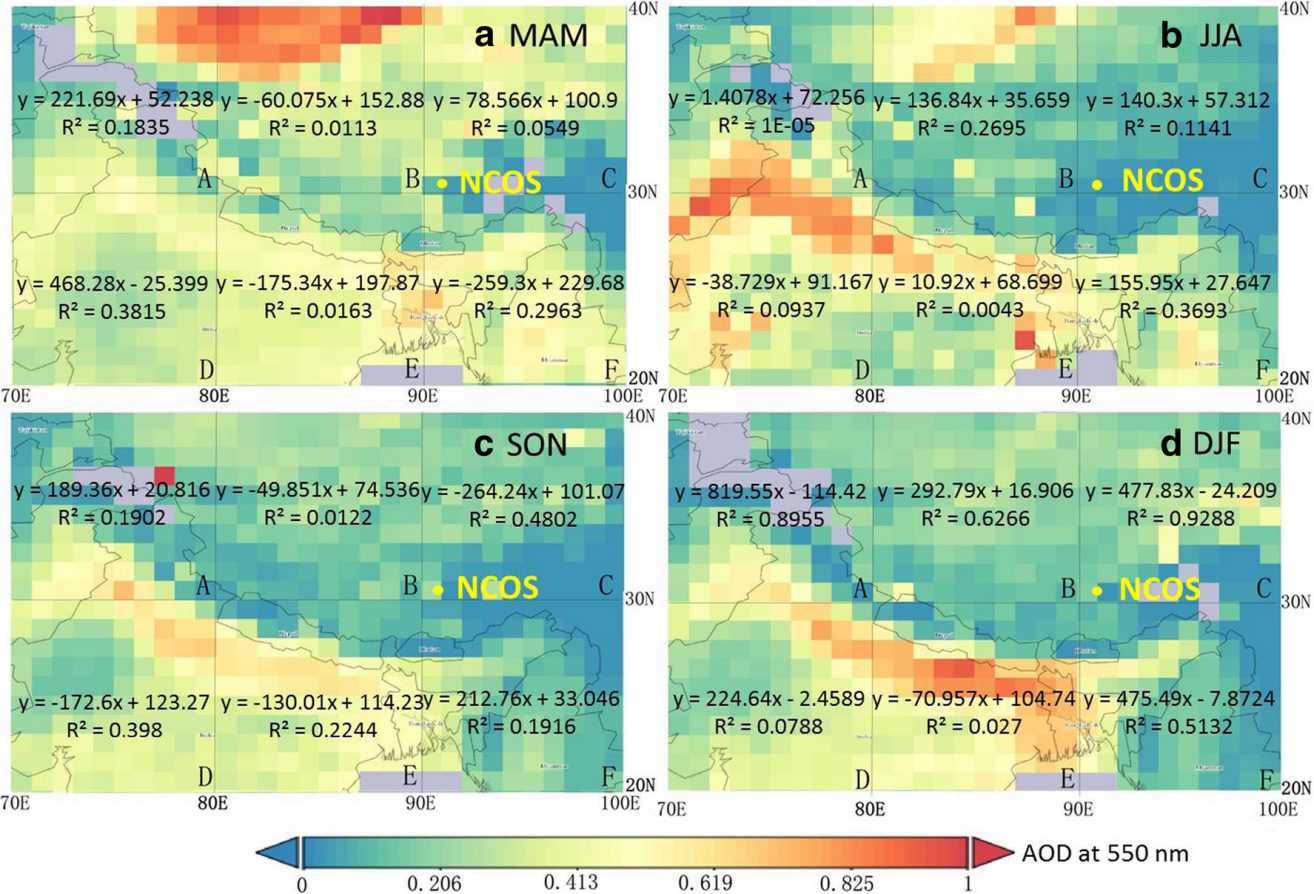
AOD distribution at TP and surrounding areas (20° N–40° N, 70° E–100° E) during 2010–2014, for which the data and plots were both generated and derived by the NASA GIOVANNI tool (https://giovanni.gsfc.nasa.gov/giovanni/). The regression equations show the relationship between AODs and BC concentrations

The seasonally area-averaged maps of the aerosol optical depth (AOD at 550 nm) including the NCOS site and its surrounding are shown in Fig. 9. Aqua MODIS dataset from October 1, 2010 through October 31, 2014 was preferred due to more reasonable passing China time of Aqua satellite (local time 13:30). The average AOD in March-April-May (MAM) is the greatest (0.38), largely attributed to emissions of mineral dust particles over the Taklimakan Desert in the northern TP, whereas the smallest average AOD (0.24) is obtained in September-October-November (SON). Large AOD value is exactly over the desert area, and it is not necessarily leading high dust loading on the AE-31 tape.

Contrast to the AODs of the TP’s surrounding areas, the AODs over the TP are generally much smaller than surrounding areas, such as the northern part of South Asia. The average daily AODs in the sub-region C in Fig. 9 (30° N–40° N, 90° E–100° E) and eBC concentrations at the NCOS have a significant co-relationship in December-January-February (DJF) (*R* ^2^ = 0.929, *α* = 0.01). The AODs in sub-area A and B also have close relations with eBC concentrations in DJF, indicating the impacts from long-distance transported pollutants by westerlies. It must be pointed out that the division of the sub-area (A–F) is very coarse and inhomogeneous, whereas it provides an approach to explore the relationship between different sources and eBC. In DJF, the correlation is relatively high between AOD in the region 30–40° N and eBC at Nam Co, possibly due to the elevated Himalaya acting as a barrier during the winter months when the PBL is thinner. Therefore, we suggested that in winters (DJF), relatively drier conditions and regional circulation could result in the accumulation of BC, majorly contributing to AOD.

This suggests the impacts from close sources on eBC variations surrounding the sampling site, consistent with the above analyses of PBLH and the diurnal-varying pattern of eBC. Previous studies have suggested that long-distance transport of pollutants from South Asia could be the dominant sources of eBC in the Nam Co region (e.g., Cong et al. 2007; Ming et al. 2010; Zhang et al. 2014). This work suggests that nearby or short-distance sources to the NCOS could also be important contributors to eBC concentrations, e.g., animal waste burning due to the local residents (Xiao et al. 2015) and fossil fuel combustion from tourists, as well as the long-distance transported pollutants by westerlies. However, previously presumed sources of eBC transported from South Asia by monsoons (Ming et al. 2010; Zhao et al. 2013) are suggested to play much weaker roles in the eBC variations observed here during 2010 through 2014.

## Conclusions

The eBC concentrations were measured online by an aethalometer at the NCOS from October 2010 to October 2014. Average daily eBC concentration was 74 ± 50 ng/m^3^, fluctuating in the range of 14–286 ng/m^3^ and reflecting a global background level. Monthly eBC concentrations were higher in February–May but lower in June–January. Meteorological conditions influenced eBC concentrations largely at seasonal scale. For example, the highest eBC concentrations (greater than 210 ng/m^3^) were more associated with the W and WSW winds smaller than 6 m/s. During pre-monsoons, eBC concentrations reached the maximum as high as over 100 ng/m^3^ attributed to lower wind speeds and less precipitations, and they kept around 50 ng/m^3^ in other seasons. The 4-year observed eBC concentrations in the Nam Co region are close to the other remote areas (Sasser et al. 2012), reflecting a global atmosphere-eBC background. The diurnal variations of eBC showed plateaus from 10:00 to 15:00 with seasonal variations, associated with local anthropogenic activities, such as residents’ burning animal waste and tourism. The PBLHs showed a co-variance with eBC concentrations, implicating nearby sources. The AODs over the sub-area (30° N–40° N, 90° E–100° E) surrounding the NCOS showed significant relationship with eBC concentrations. This also suggests that nearby or short-distance sources could be important contributors to eBC concentrations at the NCOS, different from the conclusions suggested by previous studies.
